# Face exploration, emotion recognition, and emotional enhancement of memory in relapsing-remitting multiple sclerosis

**DOI:** 10.1371/journal.pone.0319967

**Published:** 2025-04-07

**Authors:** Elisabeth Goettfried, Robert Barket, Ronen Hershman, Margarete Delazer, Michael Auer, Klaus Berek, Philipp Ellmerer, Barbara Seebacher, Harald Hegen, Franziska Di Pauli, Florian Deisenhammer, Laura Zamarian

**Affiliations:** 1 Department of Neurology, Medical University Innsbruck, Innsbruck, Austria; 2 Institute of Psychology, Leopold-Franzens University Innsbruck, Innsbruck, Austria; 3 Department of Rehabilitation Science, Clinic for Rehabilitation Muenster, Muenster, Austria; King's College Hospital NHS Trust: King's College Hospital NHS Foundation Trust, UNITED KINGDOM OF GREAT BRITAIN AND NORTHERN IRELAND

## Abstract

**Background:**

Recognizing familiar faces and identifying emotions through facial expressions are essential for social functioning. This study aimed to examine whether people with relapsing-remitting multiple sclerosis (PwMS) differ from healthy control individuals (HC) in their performance on different tasks related to facial emotion processing.

**Methods:**

In a cross-sectional controlled study, 30 PwMS and 35 HC completed a baseline neuropsychological evaluation and experimental tasks assessing visual exploration of facial stimuli through eye tracking, facial emotion recognition, and facial memory recognition. The facial stimuli displayed either a neutral expression or an emotion (happiness, fear, or disgust).

**Results:**

PwMS and HC performed comparably in facial emotion recognition. In facial memory recognition, HC were significantly more accurate in recognizing previously seen fearful faces compared to neutral faces (Wilcoxon test, *Z* = -2.26, *P* = 0.024), demonstrating emotional enhancement of memory. In contrast, PwMS did not exhibit a memory advantage for fearful faces over neutral faces (*P* > 0.05). Groups also differed in the eye-tracking task. In all but one condition (disgust), PwMS showed a significantly greater tendency to explore the eye area rather than the mouth area compared to HC.

**Conclusions:**

Changes in visual exploration and a lack of emotional enhancement of memory are observed in PwMS, who otherwise demonstrate intact facial emotion recognition. These results suggest altered emotion-cognition interactions in PwMS. Early detection of subtle changes and targeted interventions may help prevent future debilitating impairments in social functioning.

## Introduction

Skills such as the ability to recognize familiar faces or identify emotions in others through facial expressions are crucial for social functioning [1]. Difficulties in perceiving and interpreting non-verbal affective cues such as facial expressions, prosody, or bodily gestures have been linked to lower social competence, reduced social communication skills, and a poorer quality of life, significantly impacting social interactions and everyday functioning [2–4].

Emotion processing can significantly influence cognitive performance [5,6]. For example, emotional information is typically remembered better and more vividly than non-emotional information [5]. Studies using implicit memory paradigms have shown that healthy people recall fearful facial expressions better than neutral expressions, even when not explicitly instructed to do so [7]. This enhancement effect of emotions on memory is often disrupted in patients with dysfunctions of the limbic system and related structures [7]. Emotional information may also modulate attention, resulting in differences in the processing of emotionally arousing versus emotionally non-arousing (neutral) visual stimuli [6].

Multiple sclerosis (MS) is an inflammatory, demyelinating disease of the central nervous system [8]. Cognitive deficits are most commonly observed in information processing speed, episodic memory, executive functions, and attention, and are present across all MS phenotypes [9–11]. Impairments in emotion processing have also been reported [12–15]. Compared to healthy individuals, those with MS exhibit greater difficulties in tasks assessing Theory of Mind or facial emotion recognition [12]. Additionally, differences are observed in measures of alexithymia, which refers to an individual’s ability to perceive and understand their own emotions, with people with MS often scoring higher on these measures than healthy individuals [13]. Impairments in facial emotion recognition affect all individual emotions to varying degrees and are observed across all MS phenotypes, even in the early stages of the disease, with more severe impairments noted in progressive forms [12–14]. While some studies suggest a correlation between impairments in facial emotion processing and factors such as cognitive deficits, disability level, disease duration, and fatigue [16], the evidence is not uniform, with some studies failing to find such associations [17,18].

Despite the known difficulties in emotion processing among individuals with MS, the interactions between emotion and cognition remain poorly understood. To investigate this further, our study aimed to explore these interactions by utilizing both explicit and implicit paradigms of facial emotion processing. We examined attention processing and visual exploration of facial stimuli through eye tracking, as well as facial emotion recognition and the memory advantage for fearful faces compared to neutral faces in people with relapsing-remitting MS (hereafter referred to as PwMS) and healthy control participants (hereafter referred to as HC). Since the eye-tracking task involved age estimation, for which the interpretation of facial expressions is not relevant for successful task performance, we hypothesised – consistent with the findings of Liao et al. [19] – that individuals would spend time examining the upper (eyes) and lower (mouth) parts of the face for signs of aging, regardless of the emotional valence of the facial stimuli. Additionally, we expected that the enhancement effect of emotions on memory would be reflected in the finding that fearful faces are better remembered than neutral faces [7]. Whether PwMS would differ from HC in any of the facial emotion processing tasks in this study remains an open question. There is significant variability in findings across different studies: some research has reported significant changes in emotion processing already in the early stages of the disease, while other studies have not [12–14].

## Materials and methods

### Study design, setting, and participants

This was a cross-sectional controlled study conducted between June 2020 and December 2023. A total of 30 people with a confirmed diagnosis of relapsing-remitting MS, according to the 2017 revised McDonald criteria [20], and 35 HC agreed to participate in this study. PwMS were prospectively recruited as outpatients from the Department of Neurology at the Medical University of Innsbruck. HC did not report any health-associated complaints and were recruited through acquaintances or word of mouth. The inclusion criteria for both groups required participants to be over 18 years of age and to have good (corrected) near visual acuity of 20/25 (0.8) or better, as assessed by a Snellen chart. For the patient group, additional inclusion criteria were a confirmed diagnosis of relapsing-remitting MS as per the 2017 revised McDonald criteria [20], an Expanded Disability Status Scale (EDSS) [21] score of 4 or lower, no recent clinical relapse, and no current optic neuritis that could hinder participation in the eye-tracking assessment.

The exclusion criteria were as follows: eye surgery; actual optic neuritis; substance abuse; or a history of a major medical, psychiatric, or neurological disorder other than MS. We also excluded people experiencing current major depressive episodes. This information was collected through informal interviews for HC and from medical records for PwMS. All participants had an estimated verbal intelligence quotient [22] of at least 85.

### Standard protocol approvals and informed consent

The study was approved by the ethics committee of the Medical University of Innsbruck (ethical approval number: 1047/2020) and conforms to the World Medical Association Declaration of Helsinki regarding studies involving human subjects. Written informed consent was obtained from all participants prior to their involvement in the study.

### Study procedure and data collection

Participants underwent a baseline neuropsychological evaluation and experimental tasks assessing visual exploration of facial stimuli through eye tracking, facial emotion recognition, and facial memory recognition.

### Baseline neuropsychological assessment

Participants completed a baseline neuropsychological assessment that included standardized cognitive tests measuring episodic memory [23,24], verbal attention span [25], verbal working memory [25], figural fluency [26], psychomotor speed [27], cognitive flexibility [27], information processing speed [28], visuo-construction [24], and facial emotion recognition [29]. Additionally, they completed questionnaires assessing alexithymia [30] and mental health [31]. PwMS, but not HC, answered questionnaires regarding quality of life [32] and fatigue [33,34]. They also rated their current perceived fatigue severity using a visual analogue scale [35].

### Experimental tasks

#### Age estimation task (with eye tracking).

The task included 25 trials consisting of a bi-syllabic pseudo-word (e.g., KAMA) followed by a facial stimulus, while eye movements were recorded. The pseudo-words were displayed as white characters at the center of the top portion of the computer screen against a black background for 2000 msec. Facial stimuli, selected from a well-established affective stimuli database (www.emotionlab.se/kdef/) [36], were images of faces in a frontal view displaying either a neutral expression (n = 10) or emotions such as fear, disgust, or happiness (n = 5 each), with a balanced sex distribution. The pictures were aligned in terms of size, brightness, and eye position and were presented in the center of the computer screen for 5000 msec. Each picture was displayed only once, and the trials were presented in a random order that was consistent for every participant. Participants were asked to read the pseudo-word aloud and then inform the experimenter whether the displayed person appeared younger or older than 30 years. The reading task was designed to maintain participants’ attention and encourage them to refocus their gaze on the baseline (top centered) position. Answers were recorded by the experimenter but were not used for analysis purposes. Participants were unaware of the study’s purpose and were not informed that this task was part of an implicit memory paradigm.

#### Facial memory recognition task (without eye tracking).

After a delay of ca. 20 min, during which randomly selected tests from the baseline neuropsychological assessment battery were administered, participants were presented with 50 pictures. This set included the 25 facial stimuli from the age estimation task and 25 new stimuli matched for facial expression. Participants were asked to decide whether a specific picture had been shown previously. No time limit was given. Answers were recorded by the experimenter. The accuracy rate for recognizing previously seen pictures was entered into the analysis. Consistent with the study by Okruszek et al. [7], we also computed the difference in accuracy rates between fearful and neutral stimuli. A higher accuracy in recognizing previously seen fearful stimuli compared to neutral stimuli indicates an emotional enhancement of memory.

#### Facial emotion recognition task (without eye tracking).

We presented the 25 facial stimuli from the age estimation task and asked participants to identify the facial expression from seven alternatives: disgust, fear, happiness, anger, surprise, sadness, and neutrality. No time limit was given. Answers were recorded by the experimenter and analyzed as accuracy rates.

### Eye tracking

Eye movements were recorded via a TOBII Pro TX300 remote eye tracker with a sampling rate of 300 Hz. The screen resolution was 1920 × 1080 pixels. Participants were comfortably positioned approximately 64 cm from the computer screen, with their heads resting against the backrest. The TOBII Pro TX300 system is provided with head movement compensation algorithms that ensure high accuracy and precision in data collection, eliminating the need for participants to use a chinrest. We performed a 9-point calibration and visually checked its quality before starting the age estimation task. Calibration was repeated if necessary. In this study, the rate of successfully recorded eye-tracking data was at least 60%. It is important to note that the rates of successfully recorded eye-tracking data between 60% and 79% (the lowest rates in the sample) pertained to nine participants. These lower rates were associated with the use of reflective glasses, FFP2 masks worn too high on the nose, or drooping eyelids. For the analysis, the total viewing time was divided into five one-second intervals to investigate changes in visual scanning over time. The proportion of dwell time was measured for four areas: eyes, nose, mouth, and other. Consistent with the approach of Gomez-Ibanez et al. [37], our analysis focused on the difference in the proportion of dwell time between the eye and mouth areas. Positive difference scores indicate a greater predominance of visual scanning in the eye area compared to the mouth area.

### Statistics

We used SPSS software version 29.0 (IBM Corp, Chicago, IL) for our analyses, and JASP software version 0.19.2 (JASP Team, 2024) and R version 4.4.2 (R Core Team, 2023) for creating the figures. Groups were compared using the Chi-squared test and the Mann-Whitney test, where appropriate. We also computed Cohen’s *d* effect-sizes for demographic and neuropsychological data. Effect sizes can be interpreted as small (<0.50), medium (0.50-0.79), or large (≥0.80), according to Cohen’s convention. A mixed analysis of variance (ANOVA) was performed on the difference scores in the proportion of dwell time between the eye and mouth areas, with time interval (sec 1, sec 2, sec 3, sec 4, sec 5) as a within-subjects factor and group (HC, PwMS) as a between-subjects factor. This analysis was performed separately for each facial expression condition. Post-hoc contrasts were performed with Bonferroni correction. Due to a technical problem, data from one healthy participant were not recorded properly and were therefore excluded from the analysis. A Spearman rank-order correlational analysis was conducted for PwMS, examining the relationships between visual scanning measures, performance in recognition tasks, information processing speed, clinical variables, and fatigue scores. In this analysis, we focused exclusively on the fear condition. To correct for multiple correlations, we used the false discovery rate (FDR) method. Significance was set at *α*=0.05 (two-tailed), with *P*-values less than or equal to 0.05 considered significant.

## Results

Participants’ characteristics are given in Table 1. PwMS had a median EDDS score of 1.5 (min-max 0-3.5). The two groups had comparable median ages (HC: median 37.0 years, mix-max 18-64; PwMS: median 38.0 years, mix-max 21-57) and comparable median education levels (HC: median 13.0 years, mix-max 9-19; PwMS: median 12.0 years, mix-max 9-18). There were significantly more female participants in the PwMS group (n = 25; 83%) than in the HC group (n = 21; 60%). Typically, MS is more prevalent in females than in males, with a sex ratio of approximately 3:1[38], which aligns with our observations. It is important to note that, in this study, sex did not have a significant influence on performance.

**Table 1:** Participants’ characteristics.

	Max. score	PwMS (*n* = 30)	HC (*n* = 35)	*Z*-value	*P*-value	Cohen’s^d^
Female^a^		25 (83.3)	21 (60.0)	**n.a.**	**0.039** ^b^	**n.a.**
Age (years)^c^		38.0 (33.0-51.0)	37.0 (25.0-52.0)	-0.42	0.673^d^	0.06
Education (years)^c^		12.0 (11.0-15.0)	13.0 (12.0-15.0)	-1.05	0.295^d^	0.20
EDSS^c^	10	1.5 (0-2.0)	n.a.			
Disease duration (years)^c^		10.0 (5.0-18.0)	n.a.			
Current DMT at baseline						
Fingolimod^a^		3 (10.0)	n.a.			
Dimethylfumarat^a^		6 (20.0)	n.a.			
Interferon beta-1° i.m. or s.^c^.^a^		5 (16.7)	n.a.			
Natalizumab^a^		5 (16.7)	n.a.			
Other^a^		3 (10.0)	n.a.			
None^a^		8 (26.6)	n.a.			
NFI-MS physical fatigue^c^	24	9.0 (7.0-15.0)	n.a.			
NFI-MS cognitive fatigue^c^	12	5.5 (4.0-8.0)	n.a.			
Currently perceived fatigue (pre)^c^^,^^e^	10	2.0 (0-4.0)	n.a.			
Currently perceived fatigue (post)^c^^,^^e^	10	4.0 (2.0-6.5)	n.a.			
MusiQoL quality of life (index)^c^^,^^f^	100	64.8 (58.8-80.2)	n.a.			

Bold values indicate statistical significance (two-sided, *P* ≤ 0.05).

Abbreviations: PwMS =  people with multiple sclerosis; HC =  healthy control participants; n.a. =  not applicable; EDSS =  Expanded Disability Status Scale; DMT =  Disease Modifying Therapy; NFI-MS =  Neurological Fatigue Index – Multiple Sclerosis; MusiQoL =  Multiple Sclerosis International Quality of Life.

^a^Number (percentage).

^b^Chi-square test.

^c^Median (25^th^–75^th^ percentile).

^d^Mann-Whitney test.

^e^From 0 – no fatigue to 10 – extreme fatigue.

^f^Data from two PwMS are missing.

### Baseline neuropsychological assessment

Results are reported in Table 2. The median scores for both PwMS and HC fell within the average range of standardized norms across all tests. Significant group differences were observed in tests assessing verbal attention span (*P* = 0.014), verbal working memory (*P* = 0.026), and delayed recall of figural information (*P* = 0.047), with PwMS generally scoring lower than HC. Additionally, PwMS obtained significantly higher scores on the alexithymia questionnaire compared to HC (*P* = 0.017). Three PwMS (10%), but no HC, scored above the cut-off score of 60 on the alexithymia scale [30]. Cohen’s *d* indicated a medium effect size for the significant group differences. For other measures, group differences were not significant. It is important to note that the performance of PwMS on the measures where significant group differences were found did not correlate with disease severity (EDSS), disease duration, or fatigue scores (Spearman rank-order tests, all *P* > 0.05).

**Table 2:** Baseline neuropsychological assessment.

	Max. score	PwMS (*n* = 30)	HC (*n* = 35)	*Z*-value	*P*-value	Cohen’s^d^
Verbal learning (test score)^a^	75	59.5 (52.0-64.0)	61.0 (45.0-63.0)	-0.07	0.947^b^	0.12
Verbal delayed free recall (test score)^a^	15	13.5 (11.0-15.0)	12.0 (10.0-14.0)	-1.39	0.164^b^	0.20
Figural delayed free recall (test score)^a^	36	20.5 (18.0-24.0)	23.0 (19.0-28.0)	**-1.99**	**0.047** ^b^	**0.50**
Visuo-construction (test score)^a^	36	35.0 (34.0-36.0)	35.0 (34.0-36.0)	-0.56	0.574^b^	0.28
Verbal attention span (test score)^a^	12	7.0 (6.0-8.0)	9.0 (7.0-10.0)	**-2.45**	**0.014** ^b^	**0.66**
Verbal working memory (test score)^a^	12	6.0 (5.0-8.0)	7.0 (6.0-10.0)	**-2.22**	**0.026** ^d^	**0.51**
Information processing speed (correct answers)^a^^,^^d^		55.0 (50.0-65.0)	58.5 (54.5-71.0)	-1.71	0.087^d^	0.51
Psychomotor speed (time in sec)^a^		24.5 (21.0-30.0)	25.0 (20.0-33.0)	-0.46	0.645^b^	0.32
Cognitive flexibility (time in sec)^a^		58.0 (47.0-74.0)	53.0 (40.0-71.0)	-0.67	0.502^b^	0.08
Figural fluency (correct answers)^a^		28.0 (23.0-32.0)	29.0 (26.0-34.0)	-0.11	0.265^b^	0.26
Facial emotion recognition – Ekman & Friesen Test (% correct, total score)^a^^,^^c^	100	76.7 (69.2-83.3)	80.0 (73.3-86.7)	-1.43	0.152^b^	0.32
Anger (% correct)^a^^,^^c^	100	90.0 (80.0-100.0)	90.0 (80.0-100.0)	-0.50	0.619^b^	0.19
Disgust (% correct)^a^^,^^c^	100	65.0 (50.0-80.0)	80.0 (50.0-80.0)	-1.33	0.184^b^	0.35
Fear (% correct)^a^^,^^c^	100	55.0 (20.0-70.0)	60.0 (30.0-80.0)	-1.21	0.225^b^	0.33
Happiness (% correct)^a^^,^^c^	100	100.0 (100.0-100.0)	100.0 (100.0-100.0)	-0.38	0.701^b^	0.16
Sadness (% correct)^a^^,^^c^	100	80.0 (70.0-90.0)	90.0 (70.0-90.0)	-0.77	0.443^b^	0.06
Surprise (% correct)^a^^,^^c^	100	90.0 (75.0-100.0)	90.0 (80.0-100.0)	-0.63	0.530^b^	0.19
Anxiety score^a^	21	5.0 (4.0-8.0)	5.0 (3.0-6.0)	-1.45	0.146^b^	0.48
Depression score^a^	21	3.0 (1.0-5.0)	2.0 (1.0-4.0)	-1.13	0.259^b^	0.43
Alexithymia (total score)^a^	100	46.5 (35.0-53.0)	37.0 (32.0-44.0)	**-2.38**	**0.017** ^b^	**0.66**
Difficulty identifying feelings (Factor I)^a^	35	12.0 (9.0-14.0)	10.0 (8.0-12.0)	-1.52	0.129^b^	0.41
Difficulty describing feelings (Factor II)^a^	25	14.0 (8.0-19.0)	9.0 (8.0-13.0)	-1.76	0.079^b^	0.62
Externally-oriented thinking (Factor III)^a^	40	19.5 (15.0-23.0)	17.0 (14.0-20.0)	-1.84	0.066^b^	0.48

Bold values indicate statistical significance (two-sided, *P* ≤ 0.05).

Abbreviations: PwMS = people with multiple sclerosis; HC = healthy control participants.

^a^Median (25^th^–75^th^ percentile).

^b^Mann-Whitney test.

^c^Data from two PwMS are missing.

^d^This task was administered only to a subgroup of HC (n =  20).

### Experimental tasks

#### Facial memory recognition task (without eye tracking).

**Fig 1:**
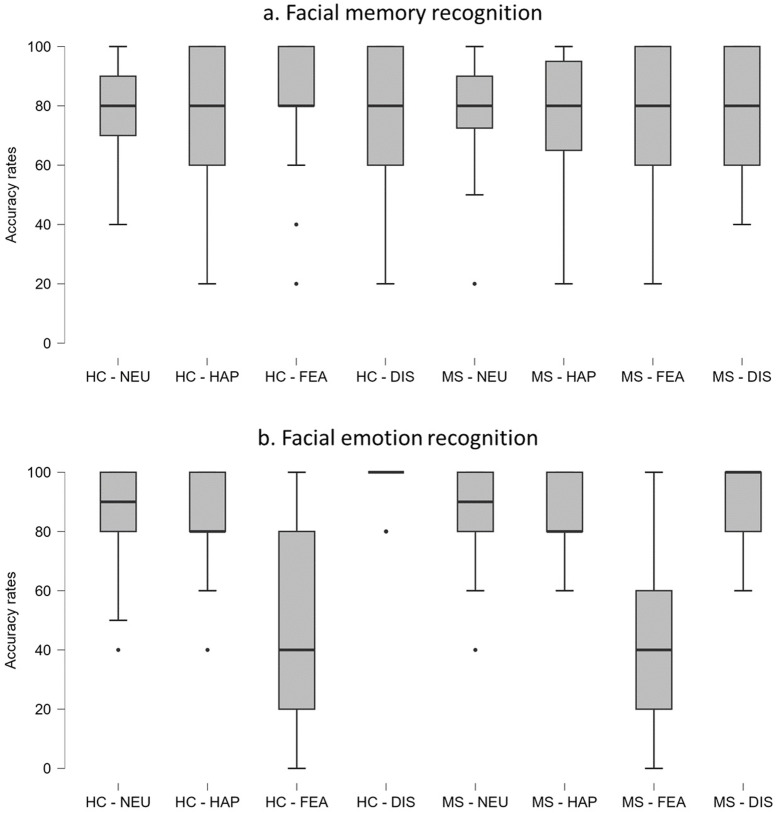
Box-plots of accuracy rates in facial memory and emotion recognition tasks (without eye tracking). Abbreviations: HC =  healthy control participants; MS =  people with multiple sclerosis; NEU =  neutrality; HAP =  happiness; FEA =  fear; DIS =  disgust.

Results are shown in Fig 1a. HC were significantly more accurate in recognizing previously seen fearful stimuli compared to other stimuli (Wilcoxon-tests, fear vs. neutrality: *Z* = -2.26, *P* = 0.024, fear vs. happiness: *Z* = -2.03, *P* = 0.042, fear vs. disgust: *Z* = -2.35, *P* = 0.019). No other significant differences were observed (all *P* > 0.05). In contrast, PwMS did not show any significant differences between facial expression conditions (all *P* > 0.05). A direct comparison of HC and PwMS across the individual facial expression conditions did not yield significant results (all *P* > 0.05).

**Fig 2:**
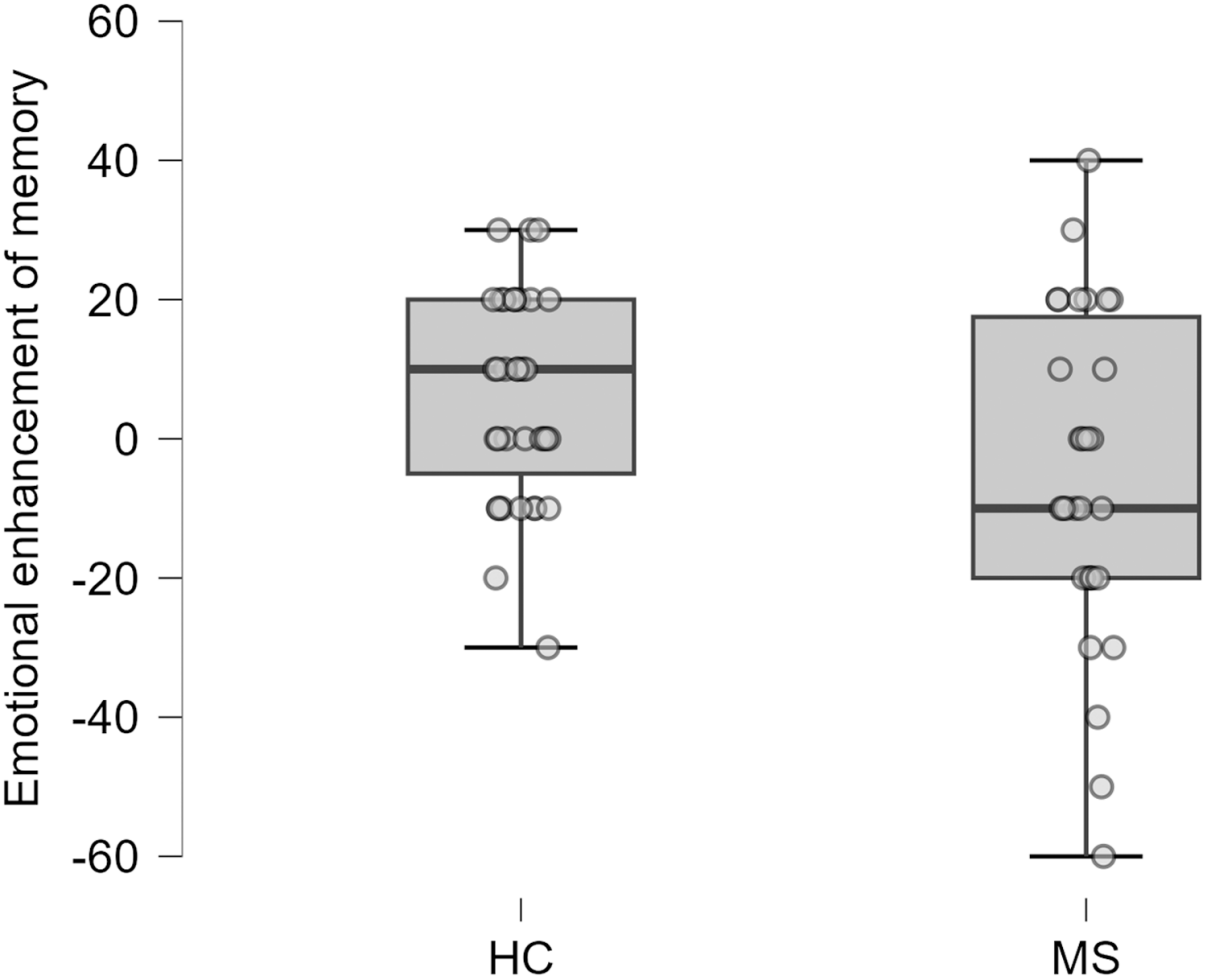
Box-plots of emotional enhancement of memory (without eye tracking). Legend. Emotional enhancement of memory is defined as the difference in accuracy rates between fearful and neutral conditions in the facial memory recognition task. Abbreviations: HC =  healthy control participants; MS =  people with multiple sclerosis.

We also compared the groups in terms of the memory enhancement effect of emotions. We found that the median difference in accuracy rates between fearful and neutral conditions was significantly smaller for PwMS than for HC (*Z* = -2.06, *P* = 0.040, Cohen’s *d* = 0.56; Fig 2). Moreover, 18 HC demonstrated higher accuracy with fearful stimuli compared to neutral stimuli (51%), while only 10 PwMS showed similar results (33%; *χ*^*2*^ = 6.65, *P* = 0.010).

#### Facial emotion recognition task (without eye tracking).

Results are shown in Fig 1b. HC were significantly less accurate in identifying fear compared to other emotions (Wilcoxon-tests, fear vs. neutrality: *Z* = -4.92, *P* < 0.001, fear vs. happiness: *Z* = -4.45, *P* < 0.001, fear vs. disgust: *Z* = -4.82, *P* < 0.001). Additionally, they were significantly less accurate in identifying neutrality and happiness compared to disgust (disgust vs. neutrality: *Z* = -3.03, *P* = 0.002, disgust vs. happiness: *Z* = -3.63, *P* < 0.001, happiness vs. neutrality: *P* = 0.695). PwMS also demonstrated lower accuracy in identifying fear compared to other emotions (fear vs. neutrality: *Z* = -4.61, *P* < 0.001, fear vs. happiness: *Z* = -4.57, *P* < 0.001, fear vs. disgust: *Z* = -4.58, *P* < 0.001). No significant differences emerged for the other comparisons (all *P* > 0.05). PwMS and HC performed comparably across each facial expression condition (all *P* > 0.05).

#### Visual exploration of facial stimuli during the age estimation task (with eye tracking).

**Fig 3:**
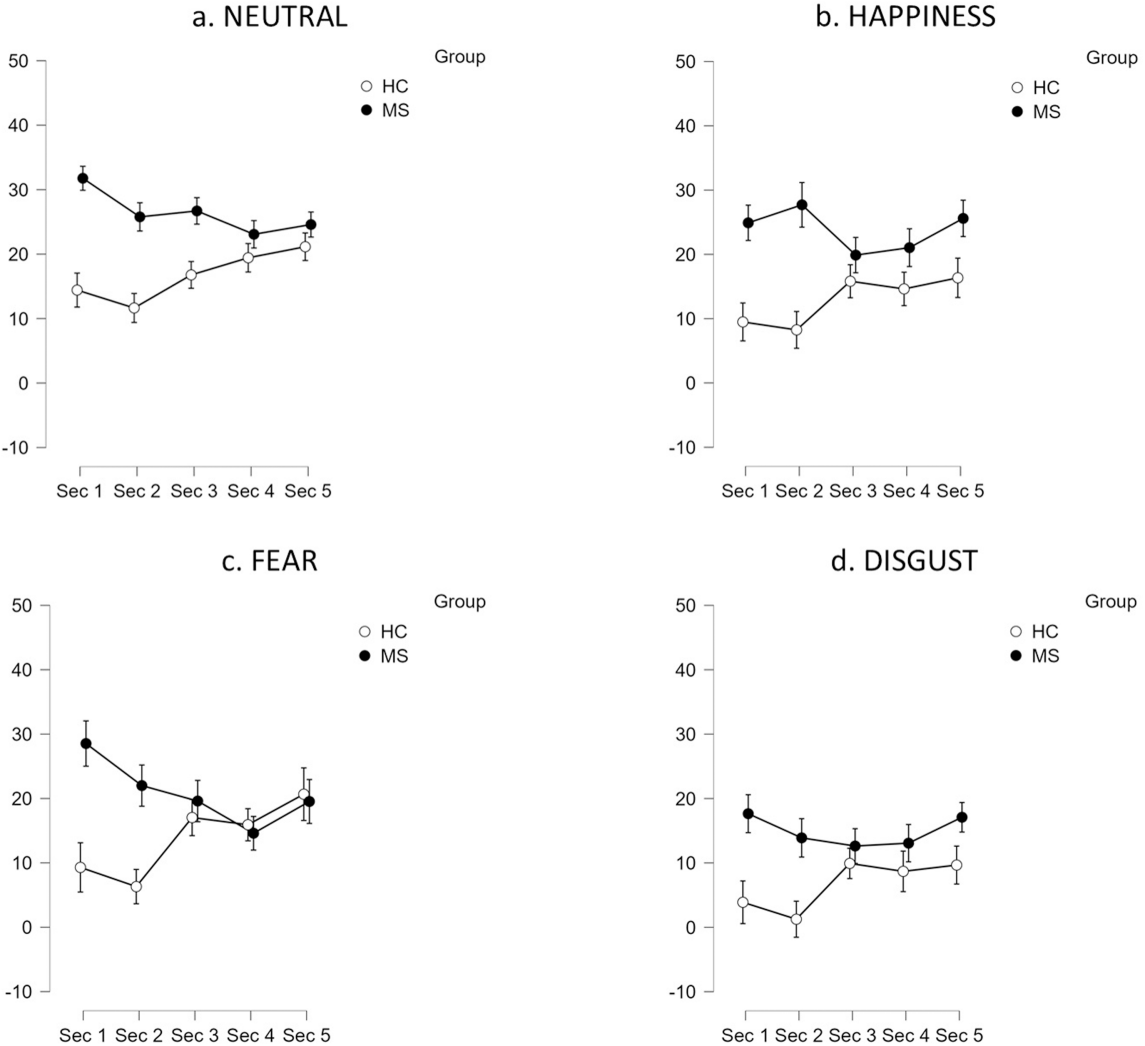
Difference scores in the proportion of dwell time between the eye and mouth areas at different time intervals (with eye tracking). Legend. Positive difference scores indicate a predominance of visual scanning in the eye area compared to the mouth area. Abbreviations: HC =  healthy control participants; MS =  people with multiple sclerosis; Sec =  second.

Results of an ANOVA carried out on difference scores in the proportion of dwell time between the eye and mouth areas showed that, across all facial expression conditions, the main effects of group and time interval were not significant (all *P* > 0.05). The interaction between time interval and group was significant for neutrality (*F*_*4,248*_ = 4.05, *P* = 0.003, *µ *_*p*_^*2*^ = 0.06; Fig 3a), happiness (*F*_*4,248*_ = 2.44, *P* = 0.047, *µ *_*p*_^*2*^ = 0.04; Fig 3b), and fear (*F*_*4,248*_ = 4.53, *P* = 0.002, *µ *_*p*_^*2*^ = 0.07; Fig 3c), but not for disgust (*P* > 0.05; Fig 3d). Post-hoc contrasts indicated significant group differences in the first (neutrality: *P* = 0.022; happiness: *P* = 0.05; fear: *P* = 0.031) and second time intervals (neutrality: *P* = 0.042; happiness: *P* = 0.004; fear: *P* = 0.016), with PwMS generally showing larger differences in the proportion of dwell time between the eye and mouth areas compared to HC. Other contrasts were not significant (all *P* > 0.05).

In summary, significant differences were observed between PwMS and HC in their visual scanning of facial stimuli. In all but one condition (disgust), PwMS exhibited marked visual exploration of the eye area compared to the mouth area throughout the entire stimulus presentation. Differently, HC explored the eye and mouth areas for nearly the same duration at the beginning of the stimulus presentation. However, from the third time interval onward, they also showed a predominance of visual scanning in the eye area, similar to what observed in PwMS.

### Correlation analysis

**Fig 4:**
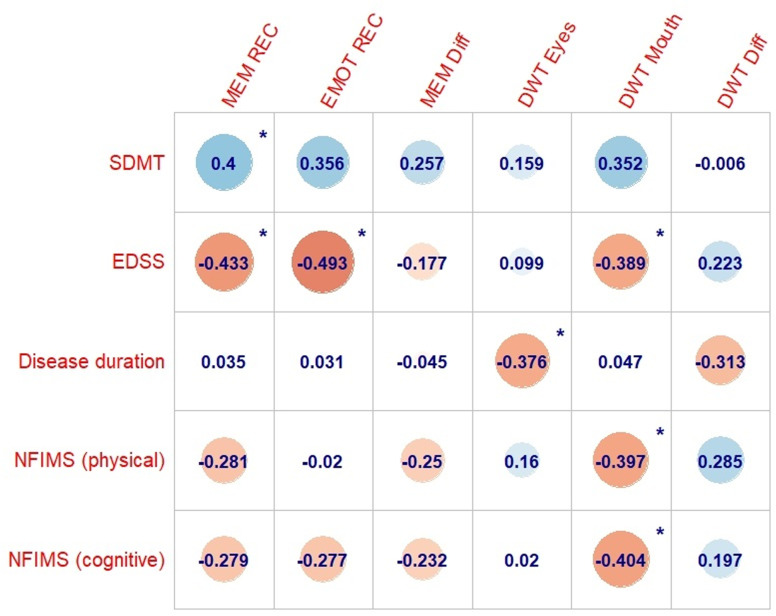
Spearman rank-order correlation coefficients (rho-values) computed for PwMS between visual scanning measures, performance in recognition tasks, information processing speed, clinical variables, and fatigue scores. (*) indicates statistical significance (two-sided, *P* ≤ 0.05, uncorrected). Abbreviations: PwMS =  people with multiple sclerosis; SDMT =  Symbol Digit Modality Test; EDSS =  Expanded Disability Status Scale; NFI-MS =  Neurological Fatigue Index – Multiple Sclerosis; MEM REC =  Accuracy in memory recognition of fearful stimuli; EMOT REC =  Accuracy in emotion recognition of fearful stimuli; MEM Diff =  Difference in accuracy rates between fearful and neutral conditions in the memory recognition task (i.e., emotional enhancement of memory); DWT Eyes =  Proportion of dwell time in the eye area (fear condition, first time interval); DWT Mouth =  Proportion of dwell time in the mouth area (fear condition, first time interval); DWT Diff =  Difference in the proportion of dwell time between the eye and mouth areas (fear condition, first time interval).

Fig 4 reports *rho*-values for PwMS. Higher accuracy in memory and emotion recognition tasks involving fearful stimuli significantly correlated with higher information processing speed and lower EDSS scores. Longer exploration of the eye and mouth areas of fearful stimuli significantly correlated with lower EDSS scores, shorter disease duration, and lower fatigue scores. These correlations remained significant after applying FDR correction. No significant correlations were found between visual scanning measures and performance in recognition tasks (data not shown).

## Discussion

In this study, we included PwMS who have minimal-to-moderate disability and found that HC, but not PwMS, exhibit a memory advantage in recognizing previously seen fearful faces compared to neutral faces. Moreover, we demonstrated that the visual scanning patterns of PwMS during an age estimation task, measured through eye tracking, differ from those of HC. In this task, participants were presented with facial stimuli depicting various emotions or neutrality, and were required to determine whether the displayed person appeared younger or older than a certain age. The emotional valence of the facial stimuli was not relevant to successful task performance. Significant differences between PwMS and HC were evident in all but one condition (disgust) during a very early phase of visual exploration. During this initial phase (the first two seconds of a five-second exploration window), HC spent almost comparable amounts of time exploring the eye and mouth areas, whereas PwMS spent less time on the lower area in favor of the upper area. In a later phase (from the third second onward), both HC and PwMS spent more time exploring the eye area than the mouth area. Since eye tracking is often used to assess visual attention, these results suggest differences in the distribution of attention and processing resources between PwMS and HC. Importantly, there was no significant difference between the groups in the time spent on the whole face (these results are not reported here), suggesting that peripheral elements such as hair or background did not attract more attention from PwMS compared to HC. Studies investigating visual scanning in PwMS during facial emotion recognition have reported heterogeneous findings [39,40]. In general, our findings, along with previously reported results [39], indicate that changes in visual scanning of facial stimuli – regardless of whether facial expressions need to be interpreted – may arise at very early stages of the disease and are not always associated with impairments in task performance. Indeed, in this study, both PwMS and HC performed comparably in the age estimation task (both groups provided similar estimates) and in the explicit facial emotion recognition task, despite their differing visual scanning patterns.

We used an age estimation task to assess the memory enhancement effect of emotions through an implicit memory paradigm. The perception and interpretation of facial characteristics, such as age or emotional expressions, are essential for social interaction [1,19]. In everyday situations, people often need to quickly gather information about others’ faces and form first impressions [19]. They may later need to remember whether they have seen a person before [1]. Previous studies have demonstrated a memory enhancement effect of emotions [5], with angry and fearful facial expressions being better remembered than neutral expressions [7]. In this study, we found that HC but not PwMS exhibit a memory advantage in recognizing previously seen fearful faces compared to neutral faces. Previous studies using verbal declarative memory tasks have shown that the performance of healthy individuals, unlike that of PwMS, is influenced by emotionally salient information [41]. This indicates that differences in the memory enhancement effect of emotions between HC and PwMS are evident for both verbal and non-verbal (facial) material. Emotional enhancement of memory has been associated with the functional integrity of a brain network that includes the amygdala, memory-related medial temporal lobe (MTL) regions such as the hippocampus, and the prefrontal cortex (PFC), particularly the ventrolateral PFC and the dorsolateral PFC [5]. Findings from different studies point to functional and structural abnormalities in MS within a cortico-subcortical network dedicated to emotion processing and, more generally, social cognition [42]. For example, in a study by Passamonti et al. [43], PwMS, compared to healthy individuals, showed enhanced activation in the ventrolateral PFC and a lack of functional connectivity between prefrontal regions (specifically, ventrolateral and medial PFC) and the amygdala when processing emotional versus neutral stimuli. Results of our study contribute to the understanding of how MS affects cognition and emotion processing by showing that the mechanisms underlying emotion-cognition interactions may be disrupted in MS, even in people with preserved facial emotion recognition, minimal-to-moderate disability, and only minor cognitive alterations. The fact that the groups did not differ in emotion recognition but showed differences in the memory enhancement effect of emotions also underscores the importance of investigating both explicit and implicit emotion processing.

In this study, PwMS showed only minor alterations in some measures of attention, memory, and alexithymia, while performing comparably to HC on the majority of neuropsychological tests, including those assessing facial emotion recognition. Findings regarding facial emotion recognition or alexithymia in MS are heterogeneous, with some studies reporting differences between PwMS and HC, while others do not [12–14]. This variability in performance, particularly in the early stages of the disease, has been discussed with reference to potential compensatory mechanisms underlying cognitive reserve and brain reserve [42].

We found that performance of PwMS in recognition tasks and their visual scanning patterns correlated with information processing speed, disease duration, physical disability, and fatigue. Previous studies have yielded mixed findings, with some indicating significant associations with cognitive and clinical variables [16,40], while others have not [17,18]. Our results provide valuable insights, suggesting that disease-related changes in PwMS may impact visual scanning, as well as emotion and memory recognition processes that are critical for social interactions and everyday functioning. Notably, we did not find a correlation between visual scanning measures and performance in recognition tasks, at least for fearful stimuli. It is possible that the observed changes in visual exploration and the lack of emotional enhancement of memory in PwMS are related to alterations in cognitive mechanisms that are at least partially independent. Future studies are needed to verify this speculation.

Different explanations can be proposed to account for the observed differences in visual scanning and the lack of emotional enhancement of memory in PwMS. MS is a neuroinflammatory, demyelinating disorder characterized by both functional and structural changes in the brain, which disrupt neural connectivity on a large scale [44]. This disruption may affect pathways responsible for visual scanning and emotion processing, potentially leading to altered visual scanning patterns and impacting how emotions influence memory. Fatigue is a common symptom of MS that can significantly impair an individual’s physical and cognitive functioning [45]. When individuals experience fatigue, their ability to effectively scan their environment or engage with complex stimuli, such as faces, may be diminished, resulting in less efficient visual and memory processing. Additionally, MS is associated with cognitive changes [9–11], which may further influence how effectively PwMS can visually scan and recall previously encountered stimuli. The significant correlations found in this study between information processing speed, disease-related variables including fatigue, and performance of PwMS on recognition tasks, as well as their visual scanning patterns, support these hypotheses. Finally, some PwMS may develop compensatory strategies to cope with their symptoms [46], which could lead to variations in visual scanning and memory performance. For example, they may rely more heavily on specific visual cues or focus on particular areas of interest, which can alter their overall scanning patterns and the way emotions influence memory. These explanations are not mutually exclusive; however, they remain speculative and require further verification.

### Limitations

One limitation of our study is that our sample consists of PwMS who have a minimal-to-moderate disability. As a result, we may have missed peculiarities related to disease severity or progression. Moreover, we did not collect response times in the behavioral tasks. Therefore, we cannot account for differences in processing speed during the execution of memory and emotion recognition tasks. Future studies could explore the differences between memory and emotion recognition tasks by employing both implicit and explicit paradigms.

## Conclusion

The results of this study make a significant contribution to previous findings [12–15] by demonstrating, for the first time, that PwMS who have otherwise intact facial emotion recognition may still exhibit alterations in visual scanning and memory processing of socially relevant non-verbal information, such as faces. In daily life, these impairments can lead to inadequate processing of visual cues and difficulties in recognizing familiar faces, which may result in misunderstandings and hinder social interactions. Our findings strongly advocate for healthcare professionals to be aware that PwMS in the early stages of the disease may experience subtle changes in some aspects of social functioning. This underscores the need to include specific neuropsychological tests that focus on visual scanning and memory processing of facial stimuli. Such assessments could facilitate the early identification of changes in social functioning among PwMS. Early detection may, in turn, provide opportunities for targeted cognitive interventions that emphasize visual exploration of facial stimuli and memory strategies, potentially preventing or mitigating future impairments in psychosocial functioning and enhancing overall quality of life.

## Supporting informations

S1 Fig Example of visual scanning patterns with a facial stimulus (fear condition) at different time intervals. (DOCX)
