# Supplementary material for: Face exploration, emotion recognition, and emotional enhancement of memory in relapsing-remitting multiple sclerosis
Source: PLoS One. 2025 Apr 7;20(4):e0319967. doi: 10.1371/journal.pone.0319967 (PMC11975382; doi:10.1371/journal.pone.0319967)
Supplement: S1 Fig — (DOCX) [file pone.0319967.s001.docx]

**Supplementary material**

S1 Figure. Example of visual scanning patterns with a facial stimulus (fear condition) at different time intervals.


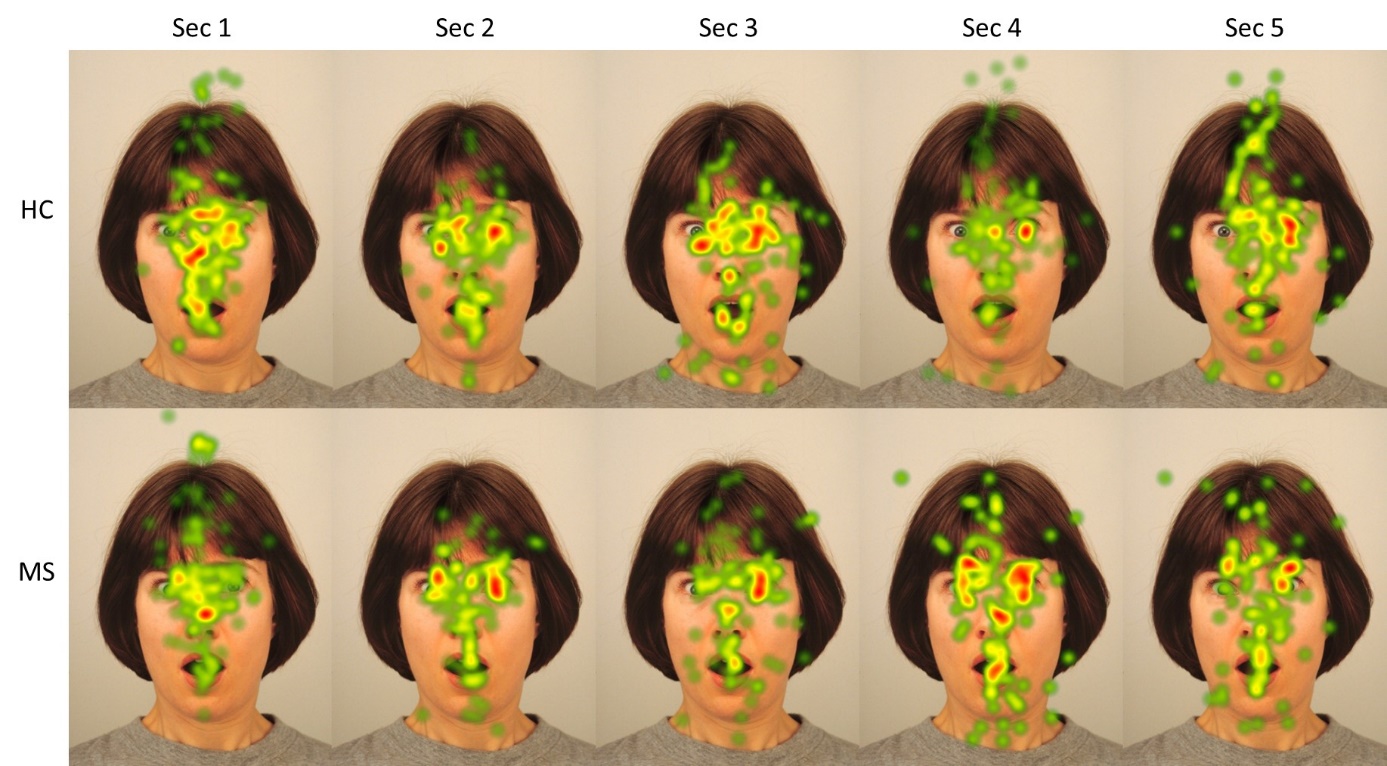


Abbreviations: HC = healthy control participants; MS = people with multiple sclerosis; Sec = second.
